# Genome Study of α-, β-, and γ-Carbonic Anhydrases from the Thermophilic Microbiome of Marine Hydrothermal Vent Ecosystems

**DOI:** 10.3390/biology12060770

**Published:** 2023-05-25

**Authors:** Mohammad Sadegh Gheibzadeh, Colleen Varaidzo Manyumwa, Özlem Tastan Bishop, Hossein Shahbani Zahiri, Seppo Parkkila, Reza Zolfaghari Emameh

**Affiliations:** 1 Department of Energy and Environmental Biotechnology, National Institute of Genetic Engineering and Biotechnology (NIGEB), Tehran 14965/161, Iran; m_gheibzadeh@nigeb.ac.ir (M.S.G.); shahbani@nigeb.ac.ir (H.S.Z.); 2 Research Unit in Bioinformatics (Rubi), Department of Biochemistry and Microbiology, Rhodes University, Grahamstown 6140, South Africa; colleen.manyumwa06@gmail.com (C.V.M.); o.tastanbishop@ru.ac.za (Ö.T.B.); 3 Faculty of Medicine and Health Technology, Tampere University, 33520 Tampere, Finland; seppo.parkkila@tuni.fi; 4 Fimlab Ltd., Tampere University Hospital, 33520 Tampere, Finland

**Keywords:** big data mining, carbonic anhydrase, extreme ecosystems, horizontal gene transfer, hydrothermal vents, mobile genetic elements, thermophilic microbiome

## Abstract

**Simple Summary:**

Hydrothermal vents are regions such as hot springs found on the seafloor in the mid-ocean and near tectonic plates. They contain fluids with highly enriched carbon dioxide, which is the central element of life on Earth. Many organisms live in this environment and can survive in extreme conditions (extremophiles), such as up to 400 °C or higher, low pH, and high pressure. All organisms need the carbonic anhydrase (CA) enzyme to handle the acid-base imbalance through the hydration of carbon dioxide and the production of bicarbonate necessary for pH homeostasis and many cellular functions. The CAs have been categorized into eight families. In this study, we focused on α-, β-, and γ-CAs from the thermophilic microbiome of marine hydrothermal vents. Microorganisms in this environment need CA to capture CO_2_, which is an important contribution to marine hydrothermal vent ecosystem functioning. Previously, we showed the transfer of β-CA gene sequences from prokaryotes to protozoans, insects, and nematodes via horizontal gene transfer (HGT). HGT is not only the transfer and movement of genetic information between organisms but is also a powerful tool in natural biodiversity. If the CA coding gene is transferred horizontally between microorganisms in hydrothermal vents, it is hypothesized that CA is essential for survival in these environments and one of the key players in the carbon cycle in the ocean.

**Abstract:**

Carbonic anhydrases (CAs) are metalloenzymes that can help organisms survive in hydrothermal vents by hydrating carbon dioxide (CO_2_). In this study, we focus on alpha (α), beta (β), and gamma (γ) CAs, which are present in the thermophilic microbiome of marine hydrothermal vents. The coding genes of these enzymes can be transferred between hydrothermal-vent organisms via horizontal gene transfer (HGT), which is an important tool in natural biodiversity. We performed big data mining and bioinformatics studies on *α-, β-,* and *γ-CA* coding genes from the thermophilic microbiome of marine hydrothermal vents. The results showed a reasonable association between thermostable α-, β-, and γ-CAs in the microbial population of the hydrothermal vents. This relationship could be due to HGT. We found evidence of HGT of α- and β-CAs between *Cycloclasticus* sp., a symbiont of *Bathymodiolus heckerae,* and an endosymbiont of *Riftia pachyptila* via Integrons. Conversely, HGT of β-CA genes from the endosymbiont Tevnia jerichonana to the endosymbiont Riftia pachyptila was detected. In addition, *Hydrogenovibrio crunogenus* SP-41 contains a *β-CA* gene on genomic islands (GIs). This gene can be transferred by HGT to *Hydrogenovibrio* sp. MA2-6, a methanotrophic endosymbiont of *Bathymodiolus azoricus*, and a methanotrophic endosymbiont of *Bathymodiolus puteoserpentis.* The endosymbiont of *R. pachyptila* has a *γ-CA* gene in the genome. If *α-* and *β-CA* coding genes have been derived from other microorganisms, such as endosymbionts of *T. jerichonana* and *Cycloclasticus* sp. as the endosymbiont of *B. heckerae*, through HGT, the theory of the necessity of thermostable CA enzymes for survival in the extreme ecosystem of hydrothermal vents is suggested and helps the conservation of microbiome natural diversity in hydrothermal vents. These harsh ecosystems, with their integral players, such as HGT and endosymbionts, significantly impact the enrichment of life on Earth and the carbon cycle in the ocean.

## 1. Introduction

Deep-sea hydrothermal vents are one of the best environments for evolutionary studies. Hydrothermal vents are regions such as hot springs found on the seafloor. These are located in the mid-ocean and near tectonic plates initially discovered in 1977 at a depth of 2.5 km around a hot spring on the Galápagos volcanic rift (spreading ridge) off the coast of Ecuador [1,2]. Based on their characteristics, deep-sea hydrothermal vents are called either black smokers or white smokers [3]. Black smokers’ fluid temperature goes up to 400 °C or above and has a low pH, but white smokers have an alkaline pH, and their temperature is approximately 40–75 °C [3]. Hydrothermal vents contain fluids with highly enriched carbon dioxide (CO_2_), which are discharged into the deep sea by these vents [4]. CO_2_ is a very stable form of carbon, the central element of life on Earth, and consists of a carbon atom covalently double-bonded to two oxygen atoms. Carbonic acid (H_2_CO_3_) is derived from the reaction of CO_2_ and water molecules, so the product is an unstable compound that spontaneously splits into bicarbonate (HCO_3_^−^) and protons (H^+^).

Many organisms live in this environment, especially bacterial and archaeal species that can survive in extreme conditions such as high temperatures and pressure. The organisms adapted to this habit are called extremophiles. All organisms need carbonic anhydrases (CAs) to handle the large amount of CO_2_ and, consequently, the related acid-base imbalance [5,6,7]. CA is the metalloenzyme that catalyzes the reversible hydration of CO_2_ to HCO_3_ and H^+^ as follows:

CO_2_ + H_2_O ↔ HCO_3_^−^ + H^+^

CAs are encoded by eight evolutionarily divergent gene families, including alpha (α), beta (β), gamma (γ), delta (δ), zeta (ζ), eta (η), theta (θ), and iota (ι) CA. α-CA has been reported in vertebrates, prokaryotes, fungi, algae, protozoa, and plants [7]. β-CA is expressed in prokaryotes, plants, fungi, protozoa, arthropods, and nematodes [8,9,10,11,12,13]. γ-CA is present in many plants, fungi, and prokaryotes. δ-CA and ζ-CA are present in marine diatoms [7,12]. η-CA was identified in the causative agent of malaria, *Plasmodium* spp., and θ-CA was identified in marine diatoms [7,14,15]. Iota(ι)-CA was recently reported to be expressed in diatoms and bacteria [16]. In this study, we focused on α-, β-, and γ-CAs from the thermophilic microbiome of marine hydrothermal vents. These metalloenzymes have an active site containing a Zn(II) metal ion cofactor [17], while Co(II) and Fe(II) can be included in α- and γ-CA, respectively [7]. The structures of α-CAs are frequently monomers and rarely dimers [18]; β-CAs are dimers, tetramers, or octamers [19]; and γ-CAs are trimers [20].

A previous study showed that β-CA gene sequences could be transferred from prokaryotes to protozoans, insects, and nematodes via HGT [21]. Additionally, the involvement of bacterial β-CA gene sequences in the gastrointestinal tract and their horizontal transfer to their host during evolution has been demonstrated [22]. HGT, also called lateral gene transfer (LGT), is the transfer and movement of genetic information between organisms and thus is differentiated from the vertical transmission of genes from parent to the next generations [23]. HGT plays a crucial role in natural biodiversity as a general mechanism [24,25], and it often causes dramatic changes in the ecological and pathogenic properties of bacterial species, thereby promoting microbial diversification and speciation [25]. HGT may occur via mobile genetic elements (MGEs) such as integrons, genomic islands (GIs), integrative conjugative elements (ICEs), transposable elements (TEs), plasmids, and phages [26,27,28,29,30]. MGEs are parts of DNA that encode enzymes and other proteins that interpose the transfer of DNA in HGT within genomes (intracellular mobility) or between bacterial cells (intercellular mobility) [26]. Intercellular transfer of DNA takes three forms in prokaryotes: transformation, conjugation, and transduction [31].

Integrons are MGEs that allow the capture and expression of exogenous genes. Integrons have three essential core features: intI, attI, and Pc [32,33]. IntI is the gene encoding an enzyme for catalyzing recombination between incoming gene cassettes called integron integrase (IntI) [32]. AttI is an integron-associated recombination site [33], and Pc is an integron-associated promoter that is expressed once a gene cassette is recombined [34]. The length of GIs is more than 10 kb, a part of a chromosome, recognized as discrete DNA segments, and can be different from closely related strains, and transposase is a primary tool for HGT through GIs [32,33,34]. Another family of MGEs is the integrative conjugative element (ICE), called the conjugative transposon. ICEs have two features: first, they are integrated into a host genome, and second, they encode a type IV secretion system (functional conjugation system) [28,35]. TEs are DNA sequences that can move from one location to another in the genome [30]. TEs fall into two classes: retrotransposons (Class I) or RNA transposons [36] and true transposons (Class II) or DNA transposons that consist of a transposase gene with two terminal inverted repeats (TIRs) on either side [30]. Additionally, insertion sequences (IS) are small MGEs that carry more than one or two transposase genes [37]. The CA genes may be transferred between organisms living in hydrothermal vents and their endosymbionts via HGT. Endosymbiotic bacteria are located in the trophosome of the host, which contains animal cells, so-called bacteriocytes [38]. For instance, one of the important living organisms living in deep-sea hydrothermal vents is the giant tubeworm *Riftia pachyptila*, which lives with its symbiont bacteria. Nitrate, oxygen, hydrogen sulfide, and inorganic carbon are taken up from the environment and it feeds its symbiotic bacteria with these substances in an organ known as the trophosome [39].

In addition, about one-third of the added carbon from atmospheric CO_2_ uptake into the ocean increases dissolved CO_2_ in seawater [40]. The accompanying acidification may reduce the seawater saturation of calcite, thus affecting marine calcifications. CA helps the concentration of inorganic carbon in the fluid from which calcium carbonate is sedimented and directly affects the calcification in some calcifiers, such as gastropods, oysters, and giant clams as well as coral calcification. The calcification can be reduced by 40%, which has been affected by high atmospheric CO_2_ levels. Even a modest impact on producing carbonate shells and skeletons may have important consequences on the global carbon cycle [41].

Microorganisms in this environment need CA to capture CO_2_, which is an important contribution to marine hydrothermal vent ecosystem functioning [42]. It has been suggested that α-CA evolution may contribute to the vulnerability to environmental changes of bivalves and their diversity [43] since HGT would create a large variability acted on by natural selection [39]. If the coding gene of this enzyme is transferred horizontally between hydrothermal vent microorganisms, it is hypothesized that CA is essential for survival and for preserving natural biodiversity in this extreme ecosystem. For this purpose, we investigated the evolutionary relationship and the possibility of HGT in the hydrothermal vent ecosystem. We conducted a large data mining and bioinformatics study focusing on the HGT of *α-, β-* and *γ-CA* genes in the microbial population of deep-sea hydrothermal vents.

## 2. Materials and Methods

### 2.1. Identification of α-, β-, and γ-CA Sequences

**Figure 1:**
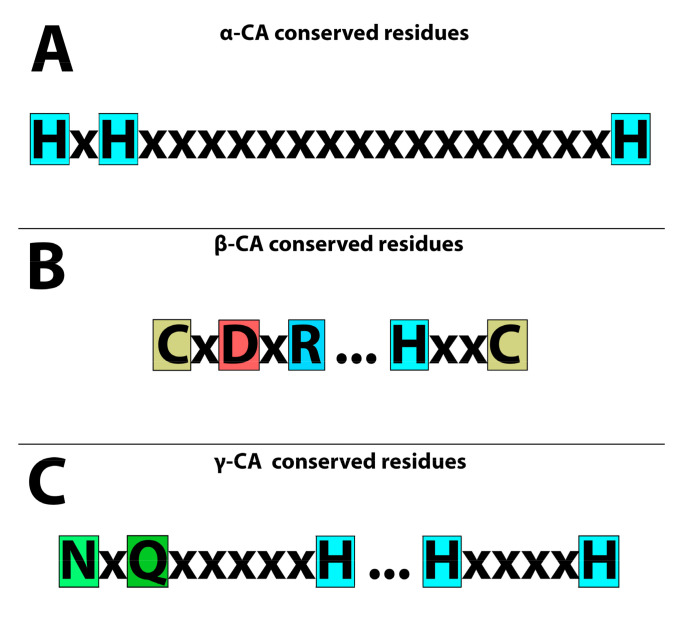
Conserved residues of CAs in the catalytic active sites. (**A**) Three “H” histidines are highly conserved in α-CAs. (**B**) Two cysteines “C”, one histidine “H”, one aspartic acid “D”, and one arginine “R” are highly conserved amino acids in β-CAs. (**C**) Three histidine residues “H”, one asparagine “N”, and one glutamine “Q” are highly conserved amino acids in γ-CAs.

We collected the names of all microbial populations from hydrothermal vents based on the literature. The protein and DNA sequences of CA candidates were retrieved from databases that were previously annotated in these databases after performing genomics and proteomics studies (Tables S1–S3). We retrieved their α-, β-, and γ-CA protein sequences from UniProt (http://www.uniprot.org/, 1 March 2023). In addition, we utilized a Position-Specific Iterated BLAST (PSI-BLAST) in the National Center for Biotechnology Information (NCBI) for two iterations to identify sequences that were homologous to the query sequences from organisms originating from hydrothermal vents. Each CA family has a defined conserved amino acid sequence to retrieve other CAs from the relevant CA family. α-CAs have three conserved histidine residues [21] (Figure 1A) that can be used as a pattern for identifying bacterial α-CAs. β-CAs have two highly conserved motifs; the first motif includes three residues of cysteine, aspartic acid, and arginine (CxDxR); the second highly conserved motif includes histidine and cysteine residues (HxxC) [21] (Figure 1B). γ-CAs have three histidine residues as well as asparagine and glutamine residues (NxQxxxxxH) and (HxxxxH) [44,45] (Figure 1C).

In addition, α-, β-, and γ-CA proteins from the microbiome of marine hydrothermal vent ecosystems with taxonomic classifications have been listed in Table S1 [46,47,48,49,50,51,52,53,54,55,56,57,58,59,60,61,62], Table S2 [63,64,65,66,67,68,69,70,71,72,73,74,75,76,77,78,79,80,81,82,83,84,85,86,87,88,89,90,91,92,93,94,95], and Table S3 [96,97,98,99,100,101,102,103,104,105,106,107,108,109,110,111,112,113,114,115,116,117,118], respectively.

Multiple sequence alignment (MSA) was performed using the Tree-based Consistency Objective Function for Alignment Evaluation (T-Coffee) [119] for the identification of conserved residues in α-, β-, and γ-CA protein sequences. Additionally, we analyzed these MSA results in Jalview2 software [120]. Then, we made a dataset for each organism (the whole genome, if available) from the NCBI database (https://www.ncbi.nlm.nih.gov/nuccore) (Access date: 1 March 2023) and apperceived the α-, β-, and γ-CA gene positions on our bacterial genomes from the Ensembl Bacteria (https://bacteria.ensembl.org) (Access date: 1 March 2023) and KEGG (https://www.genome.jp/kegg/) (Access date: 1 March 2023) databases. We annotated our integrons via Geneious prime version: 2021.0.3 software with default parameters.

### 2.2. Phylogenetic Analysis

We retrieved the Tax ID of all microbiomes from marine hydrothermal vents containing α-, β-, and γ-CA from the UniProt database (https://www.uniprot.org/taxonomy/) (Access date: 1 March 2023) and NCBI database (https://www.ncbi.nlm.nih.gov/taxonomy/) (Access date: 1 March 2023) for more accuracy. Phylogenetic trees were constructed for evolutionary study using maximum likelihood, and models with the lowest Bayesian Information Criterion (BIC) scores were considered to best describe the substitution pattern [121] via MEGA X software [122] and annotated in FigTree V1.4.4 software for all protein sequences. Then, we generated a heatmap based on the pairwise sequence identity between them using GraphPad Prism version 8.00 software for Windows (www.graphpad.com, 1 March 2023).

### 2.3. Identification of α-, β-, and γ-CA Genes on the MGEs

#### 2.3.1. Integrons

Integrons have three essential core features: intI, attI, and *Pc* [32,33,34], so we tried to find these features in our dataset. Integrons gain new genes as part of gene cassettes [123]. In addition to these features, we needed to find cassettes as simple structures consisting of a single open reading frame (ORF) bounded by a cassette-associated recombination site called a 59-base element or *attC* [124]. Gene cassettes exist in a circular free state and are integrated into *attI* [125,126]. Integron integrase mediates the integration of circular gene cassettes by site-specific recombination between *attI* and *attC* reversibly and excises [126,127,128]. For the identification of the mentioned features, we used the Integron finder. Integron Finder has two forms: a standalone program (https://github.com/gem-Pasteur/Integron_Finder) (Access date: 1 March 2023) and a web application (https://galaxy.pasteur.fr/#forms::integronfinder) (Access date: 1 March 2023). Hidden Markov model (HMM) profiles were used for the search of integron-integrase and covariance models for *attC* sites. Pattern matching was also used for other features (such as promoters and *attI* sites) [129]. In this study, we applied the web application of integron finder.

#### 2.3.2. Genomic Islands (GIs)

Prediction of GIs was studied using tools such as SIGI-HMM, IslandPath-DIMOB [130], PAI-IDA [131], and Centroid [132], based on the evaluation of sequence compositions as well as BLAST homology searches and whole-genome sequence alignment for comparative genomics methods [48]. For this purpose, we applied the IslandViewer 4 (http://www.pathogenomics.sfu.ca/islandviewer/) (Access date: 1 March 2023) database using a web server to predict and visualize genomic islands in bacterial and archaeal genomes [133]. After searching all microorganisms in this database, we retrieved their annotations and searched for *α-, β-,* and *γ-CA* genes on their GIs.

#### 2.3.3. Integrative Conjugative Elements (ICEs)

ICEs comprise the ICE integration and excision module, ICE conjugation module, and ICE regulation module, which are the main genetic modules [134]. ICEs contain integrase- and relaxase-coding genes and/or type IV secretion systems. For the identification of ICEs, we used ICEberg 2.0 (https://db-mml.sjtu.edu.cn/ICEberg/) (Access date: 1 March 2023) [135,136].

#### 2.3.4. Transposable Elements (TEs), Phages, and Plasmids

Insertion sequences (IS) and true transposons (Tn) consist of a transposase gene with two terminal inverted repeats (TIRs) on either side [30]. IS are small mobile elements that carry little more than one or two transposase genes [37]. For the identification of these elements, we used the MobileElementFinder web server (https://cge.cbs.dtu.dk/services/MobileElementFinder/) (Access date: 1 March 2023) [137]. To study phages in our datasets, we needed to find evidence of prophages. Evidence of insertion sites includes alteration of GC content and the presence of tRNA flanking the region [138]. PhageWeb (http://computationalbiology.ufpa.br/phageweb/) (Access date: 1 March 2023) was used to search for this evidence. Utilizing information from a 2018 study by Sousa, A.L.d., et al., we set options to default (BLAST options to identify 80% and six minimum of CDS) in prophage identification [139]. After that, we checked the location of our genes for the position on the chromosome or plasmid.

## 3. Results

### 3.1. Identification of α-, β-, and γ-CA and Protein Sequences

This study evaluated 83 previously isolated microorganisms in or around hydrothermal vents (Tables S1–S3). They consisted of bacteria and archaea and were classified into ten groups of bacterial species, including Alphaproteobacteria, Deltaproteobacteria, Epsilonproteobacteria, Gammaproteobacteria, Zetaproteobacteria, Aquificae, Bacilli, Deferribacteres, Deinococci, and Fusobacteria, as well as four groups for archaea, including Archaeoglobi, Methanopyri, Methanococci, and Thermococci. We retrieved 25 α-CA, 55 β-CA, and 47 γ-CA protein sequences from the UniProt database [140]. We must note that we have abbreviated microorganism names for convenience, and they are stated in the Supplementary Materials (Tables S1–S3). It is worth noting that many of these isolated species from hydrothermal vents are endosymbiotic microorganisms.

The results of the MSA for verification of α-, β-, and γ-CA protein sequences are shown in the Supplementary Materials (Figures S1–S3). Many α-CAs from the thermophilic microbiome of marine hydrothermal vents have been studied previously [42]. At first, the MSA of α-CA showed conserved residues (Figure S1) in which three conserved histidine residues (His107, His109, and His126) [21] were visible and coordinated with the Zn^2+^ metal ion cofactor in the enzyme catalytic active site [141]. Next, the MSA of β-CAs showed three conserved residues in the first highly conserved motif (CxDxR), including cysteine, aspartic acid, and arginine, with variation in the residues between them [21]. The second highly conserved motif (HxxC), which contained histidine and cysteine residues with two other residues between them, was also observed [21] (Figure S2). Finally, in the MSA of γ-CAs, we identified three histidine residues, asparagine and glutamine residues, that were highly conserved [44,45] (Figure S3).

### 3.2. Phylogenetic Analysis

**Figure 2:**
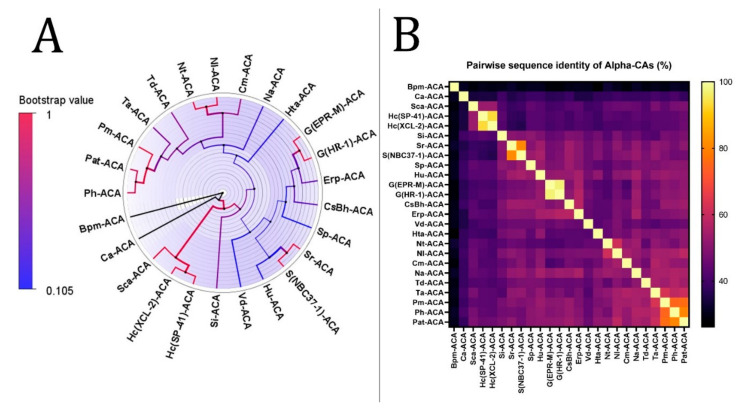
Phylogenetic analysis of α-CAs from the thermophilic microbiome of hydrothermal vents. (**A**) The tree’s branches and nodes were colored based on bootstrap values (0–1), and the bacterial CAs and archaea were highlighted with blue and orange, respectively, via FigTree V1.4.4 software. (**B**) α-CA pairwise sequence identity heatmap. The heatmap for the all-versus-all pairwise sequence identity of α-CA calculations was generated using T-Coffee MSA. Pairwise sequence identity values are colored from yellow (highest) to black (lowest).

**Figure 3:**
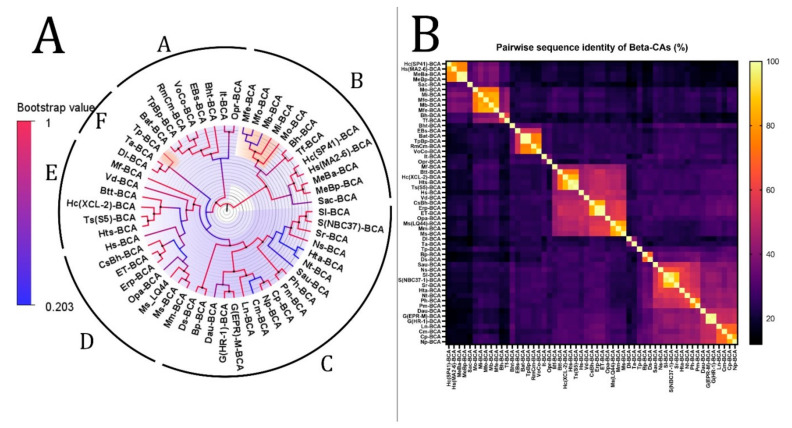
Phylogenetic analysis of β-CAs from the thermophilic microbiome of hydrothermal vents. (**A**) The tree’s branches and nodes were colored based on bootstrap values (0–1), and the bacterial CAs and archaea were highlighted with blue and orange via FigTree V1.4.4 software. (**B**) β-CA pairwise sequence identity heatmap. The heatmap for the all-versus-all pairwise sequence identity of β-CA calculations was generated using T-Coffee MSA. Pairwise sequence identity values are colored from yellow (highest) to black (lowest).

**Figure 4:**
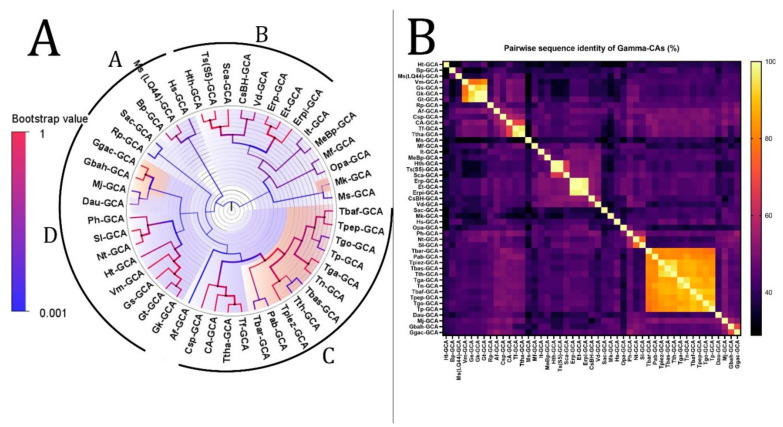
Phylogenetic analysis of γ-CAs from the thermophilic microbiome of hydrothermal vents. (**A**) The tree’s branches and nodes were colored based on bootstrap values (0–1), and the bacterial CAs and archaea were highlighted with blue and orange, respectively, via FigTree V1.4.4 software. We did not find any specific items between clades A and B or between clades C and D that can be categorized as separate clades. (**B**) γ-CA pairwise sequence identity heatmap. The heatmap for the all-versus-all pairwise sequence identity of γ-CA calculations was generated using T-Coffee MSA. Pairwise sequence identity values are colored from yellow (highest) to black (lowest).

The results of phylogenetic analysis and heatmaps of α-, β-, and γ-CAs from the thermophilic microbiome of hydrothermal vents are shown in Figure 2, Figure 3, and Figure 4, respectively. We highlighted the bacterial CAs with blue and archaea with orange. The evolutionary history was inferred using the maximum-likelihood method and the result of calculating the best model using the Le Gascuel model with discrete gamma distribution and invariable sites (LGGI) [142]. Since the phylogenetic analysis of α-CAs from the thermophilic microbiome of marine hydrothermal vents has been studied previously [42], we analyzed additional species, including *Hydrogenovibrio crunogenus* (XCL-2)*, Hydrogenovibrio crunogenus* (SP-41)*, Bacillus oceanisediminis, Sulfurivirga caldicuralii, Caldithrix abyssi*, an endosymbiont of *Riftia pachyptila* (vent Ph05), *Bathymodiolus platifrons* as a methanotrophic gill symbiont, and *Cycloclasticus* sp. as symbionts of *Bathymodiolus heckerae, Nitrosophilus alvini, Nitrosophilus labii, Sulfurimonas paralvinellae, Hydrogenimonas urashimensis, Sulfurovum indicum* and *Persephonella atlantica* (Figure 2).

The phylogenetic tree of α-CAs was performed with the highest log-likelihood (−9636.17). Initial trees for the heuristic search were obtained automatically by applying neighbor-joining and BioNJ algorithms to a matrix of pairwise distances estimated using the Jones-Taylor-Thornton (JTT) model and then selecting the topology with a superior log-likelihood value. A discrete gamma distribution was used to model evolutionary rate differences among sites (two categories (+G, parameter = 1.1935)). The rate variation model allowed some sites to evolve invariably ([+I], 3.92% sites). The tree was drawn to scale, with branch lengths measured in the number of substitutions per site. Twenty-five α-CA amino acid sequences were involved in this analysis. There were a total of 357 positions in the final dataset. The analysis revealed that there is a common ancestor between Hc(XCL-2)-ACA and Hc(SP-41)-ACA; Sr-ACA and S(NBC37-1)-ACA; G(EPR-M)-ACA and G(HR-1)-ACA; Nt-ACA and Nl-ACA; and Pat-ACA and Ph-ACA.

The phylogenetic tree of β-CAs with the highest log-likelihood (−15,146.85) is shown in Figure 3. Initial trees for the heuristic search were obtained automatically by applying neighbor-joining and BioNJ algorithms to a matrix of pairwise distances estimated using the JTT model and then selecting the topology with a superior log-likelihood value. A discrete gamma distribution was used to model evolutionary rate differences among sites (two categories (+*G*, parameter = 2.7462)). The rate variation model allowed some sites to evolve invariably ([+*I*], 0.97% sites). This analysis involved 55 β-CA amino acid sequences. There were a total of 308 positions in the final dataset. Based on bootstrap values and identity, we divided this tree (Figure 3) into six clades from A to F. The analysis revealed that there is a common ancestor between the β-CAs in each clade.

The phylogenetic tree of γ-CAs with the highest log-likelihood (−9786.77) is shown in Figure 4. The initial phylogenetic trees for the heuristic search were automatically obtained by applying Neighbor-Join and BioNJ algorithms to a matrix of pairwise distances estimated using the JTT model. The topology with a superior log-likelihood value was then selected. A discrete gamma distribution was used to model evolutionary rate differences among sites (two categories (+*G*, parameter = 1.6179)). The variation model rate allowed some sites to evolve invariably ([+*I*], 3.65% sites). Forty-seven amino acid sequences were involved in this analysis. There were a total of 219 positions in the final dataset. We divided this tree into four clades from A to D based on bootstrap values and identity, similar to the β-CA phylogenetic analysis. The analysis revealed that there is a common ancestor between the γ-CAs in each clade.

### 3.3. Identification of α-, β-, and γ-CA Genes on MGEs

#### 3.3.1. Integrons

Integrons are divided into complete integrons, In0 elements, and CALINs elements. Complete integrons have an integrase and one *attC* site or more. The In0 elements consist of an integron integrase without *attC* sites, and CALINs have two *attC* sites or more without integron integrases. After searching integron features on our dataset, we found integrons in many microorganisms, which have been mentioned in Table 1. We performed a BLAST analysis on all protein CDS (protein-coding sequences) on integrons. The results showed that only the endosymbiont of *R. pachyptila* and the endosymbiont of *Tevnia jerichonana* have CA-coding genes in their integron area.

**Figure 5:**
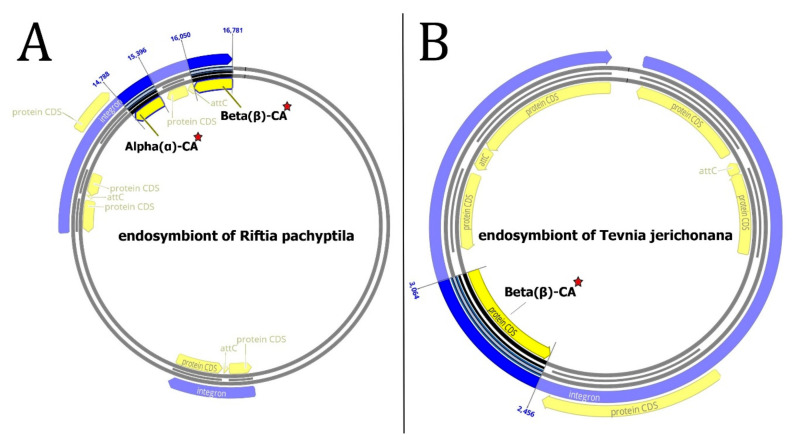
Integrons of endosymbionts of (**A**) *Riftia pachyptila* and (**B**) *Tevnia jerichonana.* The *α-* and *β-CA* genes are bolded and marked with red stars.

According to data analysis by Integron Finder, the endosymbiont of *Riftia pachyptila* contains two integrons. The first integron has one CDS, and the second has two *attC* sites; the CALIN type has six CDSs, an α-CA gene on the fourth CDS, and a β-CA gene on the sixth CDS (Figure 5A). The endosymbiont of *Tevnia jerichonana* has one integron with two *attC* sites, six CDS is CALIN type, and a β-CA gene on the fourth CDS (Figure 5B).

#### 3.3.2. Genomic Islands (GIs)

**Figure 6:**
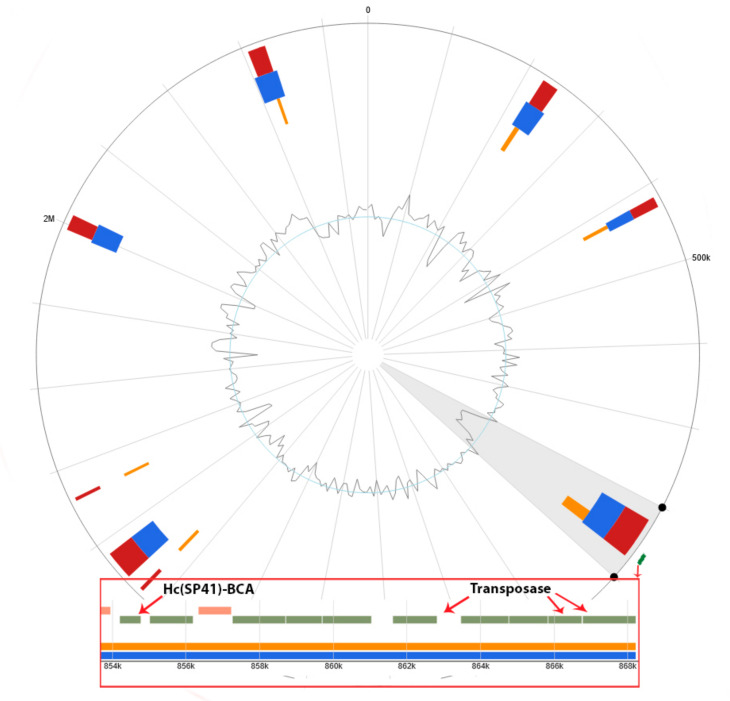
GIs of *Hydrogenovibrio crunogenus* SP-41. *Hc(SP41)-BCA* and transposase genes are marked green and shown in the red box. The GC content is visible at the center of the figure.

According to the IslandViewer 4 (http://www.pathogenomics.sfu.ca/islandviewer/) (Access date: 1 March 2023) database, 25 out of 83 of our microorganisms have GIs, and only one of the *Hydrogenovibrio crunogenus* SP-41 GIs carries a *β-CA* gene (*Hc(SP41)-BCA*) (UniProt ID: Q31FD6) and three transposase genes that are primary tools for HGT [143] (Figure 6). This GI is predicted by SIGI-HMM [144] and IslandPath-DIMOB methods [130]. However, the HGT of β-CA genes with GIs between prokaryotes and protists was previously studied [22].

#### 3.3.3. Integrative Conjugative Elements (ICEs), Transposable Elements (TEs), Phages, and Plasmids

According to ICEberg 2.0 (https://db-mml.sjtu.edu.cn/ICEberg/) (Access date: 1 March 2023) and MobileElementFinder web server results, we did not find any α-, β-, and γ-CA genes on the ICEs and TEs. Additionally, using PhageWeb (https://github.com/phagewebufpa/API (Access date: 25 April 2019), we did not find any evidence supporting the transfer of α-, β-, and γ-CA genes via phages. Based on the details of our dataset, CA genes were not located on the plasmids from the thermophilic microbiome of hydrothermal vents, and all genes were found on the chromosomes.

## 4. Discussion

The evolutionary process in hydrothermal vent ecosystems and the role of viruses in the biodiversity in this harsh environment have been studied previously. A study performed by Cheng et al. [145] revealed that bacteriophages are the most predominant viruses across the global hydrothermal vents, while single-stranded DNA viruses, including Microviridae and small eukaryotic viruses, have been located in the next steps. The metagenomics analysis showed that this virome plays a crucial role in the evolution and biodiversity of the microbiome of hydrothermal vents, especially Gammaproteobacteria and Campylobacterota [145]. Although the bacteriophages have no role in the HGT of CA genes in the hydrothermal ecosystems, our previous studies showed the HGT of *β-CA* genes from prokaryotic endosymbionts to their protozoan, insects, and nematodes hosts. In addition, the genomic islands have been shown to have a potential role in the HGT of *β-CA* genes from ancestral prokaryotes to protists. Since then, no further study has been performed on the HGT of CA genes. Since hydrothermal vent ecosystems have been reported as potent environments for HGT and biodiversity, these harsh deep-sea fissures were studied.

According to the heatmap and phylogenetic analysis (Figure 2) of α-CAs, Bpm-ACA and Ca-ACA showed no significant relationship with the other α-CAs. Hc(XLC-2)ACA, Hc(SP41)-ACA, and Sca-ACA clustered together with branch bootstrap values of 1.00, showing significant relationships. Additionally, G(HR-1)-ACA and G(EPR-M)-ACA had a branch bootstrap value of 1.00, indicating a robust evolutionary relationship similar to that between Sr-ACA and S(NBC37-1)-ACA, whose bootstrap value was also 1.00. Similar to a previous study, the branch for Pm-ACA and Ph-ACA was observed to have a high bootstrap value of 0.99. A high branch bootstrap value of 0.86 was observed for CsBh-ACA and Erp-ACA. It is necessary to mention that all the α-CAs above belong to the Proteobacteria phylum except for Pm-ACA and Ph-ACA, which belong to the Aquificae phylum. According to the heatmap and phylogenetic analysis (Figure 3) of β-CAs, in clade A, Opr-BCA and It-BCA have poor relationships with other clade members, showing a branch bootstrap value of 0.37. All members of clade B have the same root, but Mfe-BCA, Mfo-BCA, and Mb-BCA have poor relationships with other clade members. In clade C, a significant relationship between Sr-BCA, S(NBC37)-BCA, and Si-BCA showed a branch bootstrap value of 1.00, in which pairwise sequence identities of more than 88.6% were revealed. Although these three cases with a branch bootstrap value of 0.92 have a significant relationship with Ns-BCA, they have a poor relationship with other members of clade C. In clade D, a relationship between Erp-BCA, Et-BCA, and CsBh-BCA was observed with a 1.00 branch bootstrap value, in which a pairwise sequence identity of more than 83.5% was observed for all three. In clade E, the cluster containing Hts-BCA, Ts-BCA, Hc(XCL-2) –BCA, and Btt-BCA was observed with a branch bootstrap value of 1.00. Clade F with a 0.3 branch bootstrap value did not show a good relationship with other clades, while Di-BCA and Ta-BCA have the same root as Tp-BCA, a member of archaea. According to the heatmap and phylogenetic analysis of γ-CAs (Figure 4), clades A and B, with branch bootstrap values of 0.02 and 0.001, respectively, have a very poor relationship with other clades, including Erpi-GCA, Erp-GCA, and ET-GCA with different branch bootstrap values of more than 0.98 and a pairwise sequence identity value of more than 97.78, which have a significant relationship together. In addition, a meaningful relationship was observed for Hts-GCA, Ts(S5)-GCA, and Sca-GCA with a branch bootstrap value of 0.99. In clade C, archaea and bacteria have the same root, and according to the heatmap, all archaea have high pairwise sequence identity values. In clade D, Gbah-GCA and Ggac-GCA have a good relationship with a branch bootstrap value of 0.99 and a pairwise sequence identity value of 69.18. According to the heatmap of γ-CA (Figure 4B) in clade D, Gs-GCA with a branch bootstrap value of 0.99 and a pairwise sequence identity value of 94.29 had a significant relationship with Gk-GCA.

CALIN elements (Table 1) might have arisen from a missing integrase in a previously complete integron. The *α-* and *β-*CA genes from CALIN may be cut by the integron-integrase and reinserted in the integron at an *attI* site. Since the stable circular form of CALINs can survive in the environment, these genetic elements can be taken up by transformable bacteria through a transformation mechanism [146]. On the other hand, integrons often capture cassettes from CALIN elements [129], so the *α-* and *β-*CA genes can be derived from different microorganisms or transferred to other hosts. According to the phylogenetic trees of α- and β-CA (Figure 2A and Figure 3A), Erp-ACA has the highest relationship with CsBh-ACA, with a bootstrap value of 0.40 and a pairwise sequence identity value of 57.61, which is a weak relationship. In addition, it has a relatively weak relationship with G(EPR-M)-ACA and G(HR-1)-ACA twins with a bootstrap value of 0.44 and pairwise sequence identity values of 55.19 and 57.39, respectively. The β-CAs in clades A and B, with 0.02 and 0.001 branch bootstrap values, respectively, did not have a good relationship with other clades. At the same time, Erp-BCA is the highest related compound to Et-BCA and CsBh-BCA, with a 1.00 branch bootstrap value and pairwise sequence identity of 99 and 100, respectively. Moreover, the *β-*CA gene (*Et-BCA*) from the endosymbiont of *T. jerichonana* is related the highest to Erp-BCA and CsBh-BCA, with a branch bootstrap value of 1.00 and pairwise sequence identity of 99 and 84, respectively, which indicates the possibility of horizontal gene transfer of *β-*CA coding genes in these microorganisms.

It should be noted that inorganic carbon from CO_2_ is first obtained from the environment via diffusion through the plume, a branchial organ [147]. Next, CO_2_ is transformed to HCO_3_^−^ and transported to trophosome cells, particularly bicarbonate, at the surrounding branchial plume interface. Then, HCO_3_^−^ is transformed to CO_2_ on the body fluids and bacterial cells [148] and adhered via the bacterial symbiont enzyme RuBisCO form II. In the arginine biosynthesis and pyrimidine pathways, carbamylphosphate synthetase uses inorganic HCO_3_^−^ to start the biosynthesis process. Since the metabolic relationship between *R. pachyptila* and its endosymbiont is vital for the survival of each organism, this issue can explain the cause and importance of HGT of CA in these organisms. Furthermore, *R. pachyptila* contains an *α-*CA gene [149] with UniProt ID: Q8MPH8, which is not similar to Erp-ACA.

Additionally, *T. jerichonana* has no reported CA family. Identification of the *β-*CA gene beside three transposase genes on one of the GIs of *H. crunogenus* SP-41 could lead to the theory that this gene may be transferred with plasmids and phages or occur through transposon accumulation in recombination sites. Experimental studies have suggested the release of about 1.5 billion symbionts from dead tubeworm clumps into the environment [47], which provides the opportunity for the spread and HGT of CA genes in the environment and preparing the biodiversity condition.

In addition to the β-CA phylogenetic tree, the heatmap showed that the Hc(SP41)-BCA in clade B is closely related to Hs(MA2-6)–BCA with a branch bootstrap value of 0.99 and a pairwise sequence identity value of 82.9. In addition, MeBa-BCA and MeBp-BCA showed a close relationship with Hc(SP41)-BCA with branch bootstrap values of 1.0 and pairwise sequence identity values of 76.56 for both cases. The HGT of hydrogenase-coding genes between *H. crunogenus* SP-41 and *H. crunogenus* XCL-2 was studied previously [150]; however, in this study, *H. crunogenus* SP-41 (*Hc(SP41)-BCA*) had no HGT relationship with *H. crunogenus* XCL-2. *R. pachyptila* has cytosolic α-CA in the trophosome. Although these organisms need secretory CA for their physiological needs and use Erp-ACA, this theory must be experimentally studied.

The significance of this study revealed that there is an evolutionary relationship between Hc(XLC-2)ACA, Hc(SP41)-ACA, and Sca-ACA; G(HR-1)-ACA and G(EPR-M)-ACA; Sr-ACA and S(NBC37-1)-ACA; Pm-ACA and Ph-ACA; and CsBh-ACA and Erp-ACA in α-CAs. In addition, there is an evolutionary relationship between Sr-BCA, S(NBC37)-BCA, and Si-BCA; Erp-BCA, Et-BCA, and CsBh-BCA; and Hts-BCA, Ts-BCA, Hc(XCL-2) –BCA, and Btt-BCA in β-CAs. Additionally, there is an evolutionary relationship between Erpi-GCA, Erp-GCA, and ET-GCA; Hts-GCA, Ts(S5)-GCA, and Sca-GCA; Gbah-GCA and Ggac-GCA; and Gs-GCA and Gk-GCA in γ-CAs.

Elevated CO_2_ pressure in seawater can affect marine organisms by disrupting acid-base physiology and decreasing mineralization rates (affecting calcium carbonate saturation and calcification). Ocean uptake of anthropogenic CO_2_ and associated changes in seawater chemistry adversely affect biodiversity, other ecosystem processes, and the global carbon cycle [151]. The HGT and distribution of CA genes in the hydrothermal vent area may also help the survival and diversity of the organisms in this environment.

## 5. Conclusions

According to the results of this big data mining and bioinformatics study, α-, β-, and γ-CAs from the thermophilic microbiome of marine hydrothermal vents have a reasonable evolutionary relationship. The *α-*, *β-*, and *γ-CA* genes can be transferred to other microorganism habitats in hydrothermal vents via HGT and cause natural biodiversity in this extreme ecosystem. Given the presence of an integron with an integrase coding gene in the *Cycloclasticus* sp. symbiont of *Bathymodiolus heckerae,* it is highly possible that the *α-*CA coding gene is transferred between *Cycloclasticus* sp. as the symbiont of *B. heckerae* and endosymbiont of *Riftia pachyptila*. This evolutionary phenomenon can also be applied to *β-CA*-coding genes.

According to the *β-*CA gene on the endosymbiont of *T. jerichonana* and the endosymbiont of *R. pachyptila* and the evolutionary relationship between them, the HGT of the *β-*CA gene from the endosymbiont of *T. jerichonana* to the endosymbiont of *R. pachyptila* and conversely is highly possible. In addition, the endosymbiont of *R. pachyptila* has a *γ-*CA gene on the chromosome; if *α-* and *β-*CA coding genes are derived from other microorganisms, such as the endosymbiont of *T. jerichonana* and *Cycloclasticus* sp. as the symbiont of *B. heckerae,* the theory of the necessity of the CA enzyme for survival in this extreme ecosystem and its effect on preserved natural biodiversity is proposed. Despite the presence of the *α-*CA gene in *R. pachyptila* and the *α-*, *β*-, and *γ-*CA genes in its endosymbiont, this theory is suggested for this giant marine worm. Therefore, the prokaryotic endosymbionts of mussels and giant marine worms have evolutionary relationships through HGT. With more focus on the HGT phenomenon, endosymbionts are integral parts of natural biodiversity and ecosystem functioning of marine hydrothermal vents.

## Figures and Tables

**Table 1:** Integrons in the thermophilic microbiome from hydrothermal vents.

Microorganisms	Integron Type	Integrase	CA Gene
*Cycloclasticus sp.* symbiont of *Bathymodiolus heckerae* Endosymbiont of *Riftia pachyptila* (vent Ph05) *Sulfurovum* sp. NBC37-1	In0	Intersection tyr intI	-
	Integron 1: CALIN Integron 2: CALIN	-	*α-CA* *β-CA*
	Integron 1: CALIN Integron 2: CALIN	-	-
*Caldithrix abyssi* *Hydrogenovibrio crunogenus* SP-41	CALIN	-	-
	Integron 1: CALIN Integron 2: CALIN Integron 3: Complete Integron 4: CALIN	Intersection tyr intI	-
*Thiomicrospira crunogena* XCL-2 *Bathymodiolus thermophilus* thioautotrophic gill symbiont Endosymbiont of *Tevnia jerichonana* *Halomonas sulfidaeris* strain SST4	Integron 1: CALIN Integron 2: CALIN	-	-
	Integron 1: CALIN Integron 2: CALIN Integron 3: CALIN	-	-
	CALIN	-	*β-CA*
	CALIN	-	-
*Marinobacter* sp. LQ44	Integron 1: CALIN Integron 2: In0	Intersection tyr intI	-
*Sulfurimonas autotrophica*	Integron 1: In0 Integron 2: CALIN	Intersection tyr intI	-
Endosymbiont of *Bathymodiolus septemdierum*	Integron 1: CALIN Integron 2: CALIN Integron 3: In0 Integron 4: CALIN Integron 5: CALIN	Intersection tyr intI	-
*Hydrogenovibrio thermophilus* *Thermococcus barophilus* strain CH5	Complete	Intersection tyr intI	-
	CALIN	-	-
*Cycloclasticus sp.* symbiont of *Bathymodiolus heckerae*	In0	Intersection tyr intI	-
Endosymbiont of *Riftia pachyptila* (vent Ph05)	Integron 1: CALIN Integron 2: CALIN	-	*α-CA* *β-CA*
*Sulfurovum* sp. NBC37-1	Integron 1: CALIN Integron 2: CALIN	-	-
*Caldithrix abyssi*	CALIN	-	-
*Hydrogenovibrio crunogenus* SP-41	Integron 1: CALIN Integron 2: CALIN Integron 3: Complete Integron 4: CALIN	Intersection tyr intI	-
*Thiomicrospira crunogena* XCL-2	Integron 1: CALIN Integron 2: CALIN	-	-
*Bathymodiolus thermophilus* thioautotrophic gill symbiont	Integron 1: CALIN Integron 2: CALIN Integron 3: CALIN	-	-
Endosymbiont of *Tevnia jerichonana*	CALIN	-	*β-CA*
*Halomonas sulfidaeris* strain SST4	CALIN	-	-
*Marinobacter* sp. LQ44	Integron 1: CALIN Integron 2: In0	Intersection tyr intI	-
*Sulfurimonas autotrophica*	Integron 1: In0 Integron 2: CALIN	Intersection tyr intI	-
Endosymbiont of *Bathymodiolus septemdierum*	Integron 1: CALIN Integron 2: CALIN Integron 3: In0 Integron 4: CALIN Integron 5: CALIN	Intersection tyr intI	-
*Hydrogenovibrio thermophilus*	Complete	Intersection tyr intI	-
*Thermococcus barophilus* strain CH5	CALIN	-	-

## Data Availability

Not applicable.

## Supplementary Materials

The following supporting information can be downloaded at: https://www.mdpi.com/article/10.3390/biology12060770/s1. Figure S1, Multiple Sequence Alignment of α-CA from the thermophilic microbiome of hydrothermal vents; Figure S2, Multiple Sequence Alignment of β-CAs from the thermophilic microbiome of hydrothermal vents; Figure S3, Multiple Sequence Alignment of γ-CAs from the thermophilic microbiome of hydrothermal vents; Table S1, α-CA proteins from the microbiome of marine hydrothermal vent ecosystems with taxonomic classifications; Table S2, β-CA proteins from the microbiome of marine hydrothermal vent ecosystems with taxonomic classifications; Table S3, γ-CA proteins from the microbiome of marine hydrothermal vent ecosystems with taxonomic classifications.
