# Supplementary figures and images for: Genome Study of α-, β-, and γ-Carbonic Anhydrases from the Thermophilic Microbiome of Marine Hydrothermal Vent Ecosystems
Source: Biology (Basel). 2023 May 25;12(6):770. doi: 10.3390/biology12060770 (PMC10295459; doi:10.3390/biology12060770)

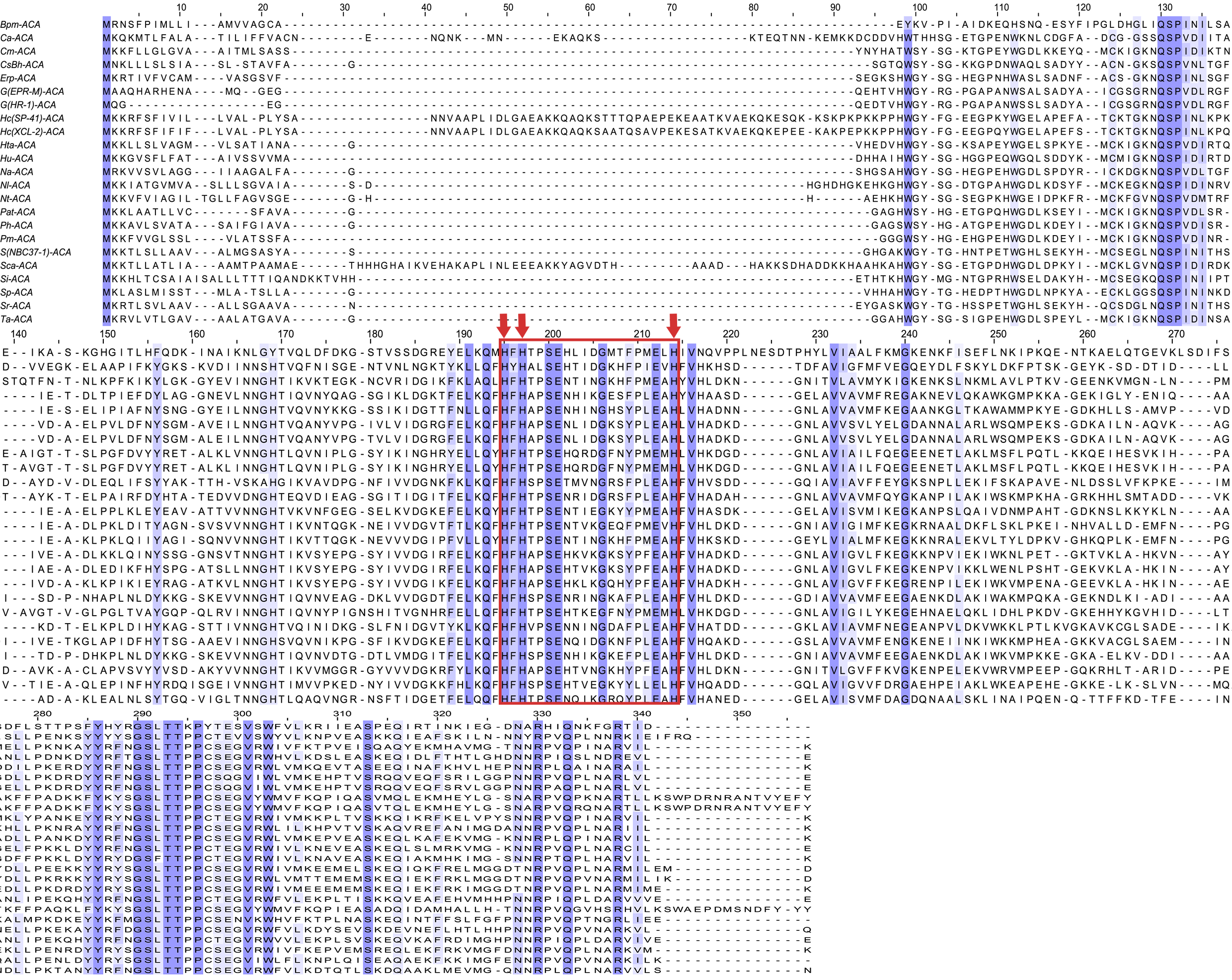

Supplement: Supplementary file 1 [file biology-12-00770-s001.zip › Figure S1.png]

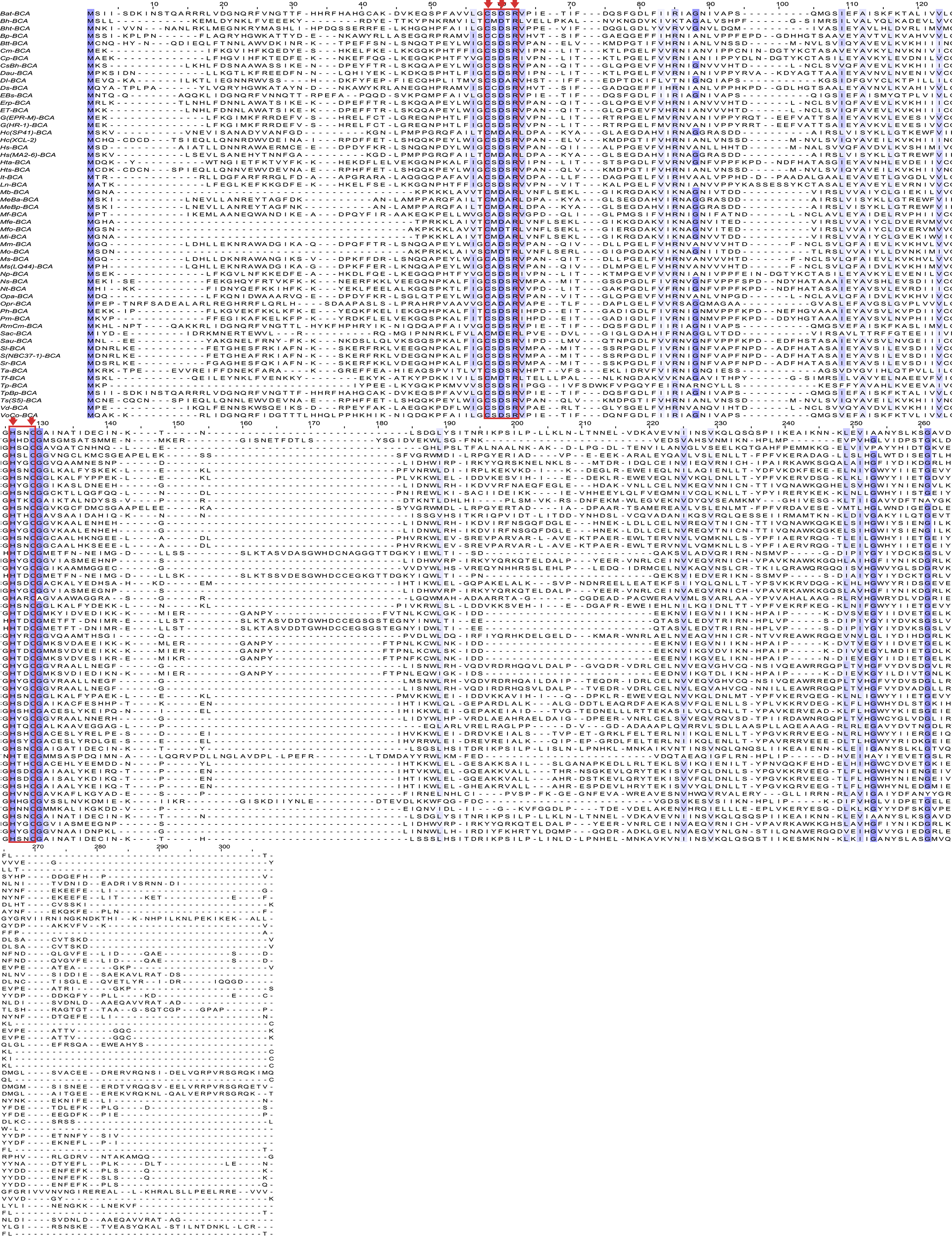

Supplement: Supplementary file 1 [file biology-12-00770-s001.zip › Figure S2.png]

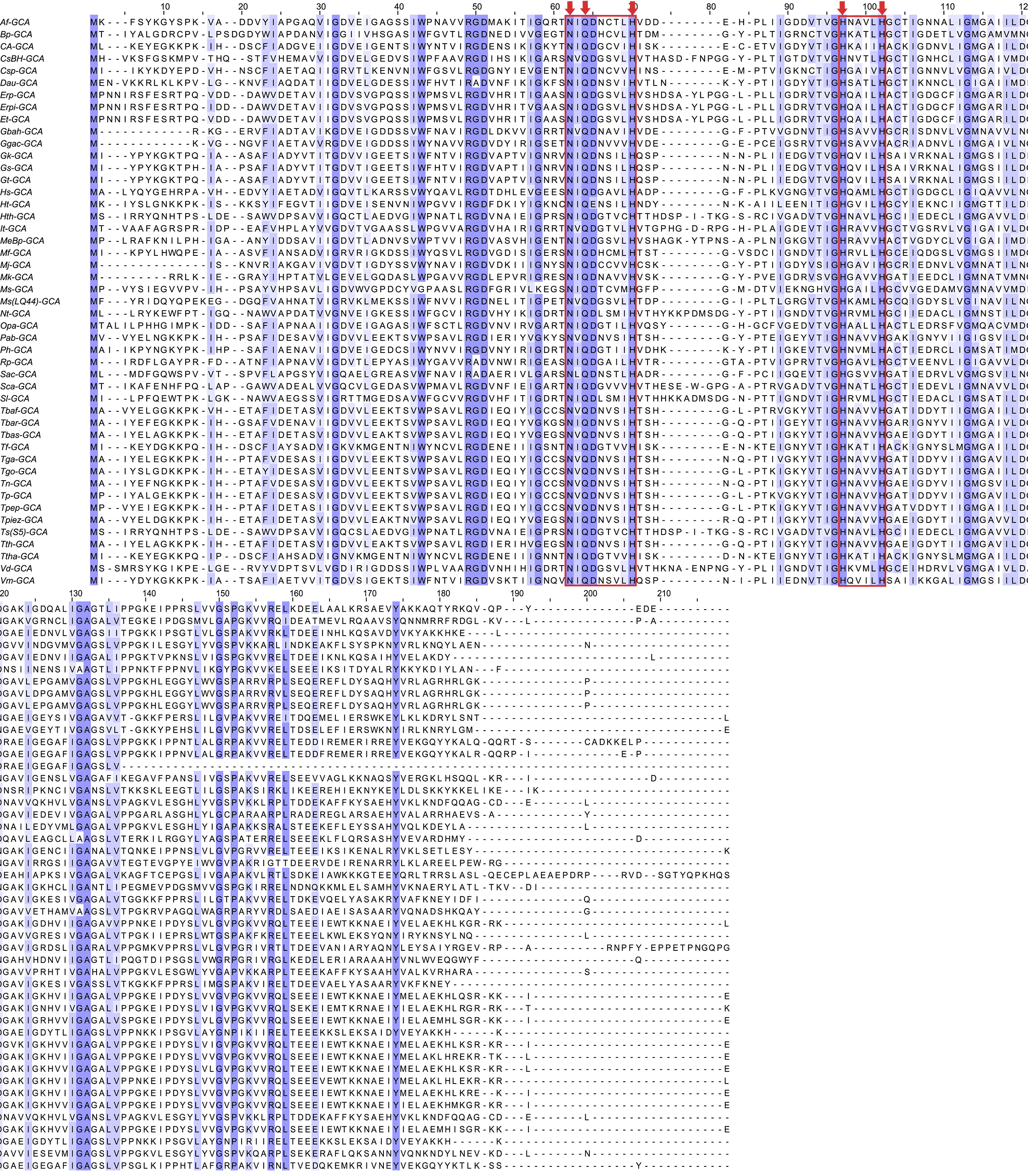

Supplement: Supplementary file 1 [file biology-12-00770-s001.zip › Figure S3.png]
